# Clear cell adenocarcinoma of the cervix, a rare entity of young females: A case report

**DOI:** 10.15190/d.2025.4

**Published:** 2025-03-31

**Authors:** Paramita Paul, Paramita Rudra Pal, Sadaf Hayat

**Affiliations:** ^1^Department of Oncopathology; Homi Bhabha Cancer Hospital & Mahamana Pandit Madan Mohan Malviya Cancer Centre (Tata Memorial Centre); Homi Bhabha National Institute (HBNI), Varanasi, Uttar Pradesh-221005, India

**Keywords:** Clear cell adenocarcinoma, cervix, diethyl-stilbesterol, histopathology.

## Abstract

Clear cell adenocarcinoma of the cervix is a rare neoplasm that accounts for about 4-9% of all uterine cervical adenocarcinoma. It was first reported in young women with in-utero exposure of diethylstilbesterol (DES). However, many cases have been reported post-ban of DES that are not associated with diethylstilbesterol (DES). Here, we are reporting a case of clear cell adenocarcinoma in an 18-year-old woman without any exposure history of DES. An exophytic mass arising from the anterior lip was found in MRI. Biopsy from the mass was diagnosed as Clear cell adenocarcinoma of the cervix based on histopathological and immunohistochemical findings. The patient then underwent a total hysterectomy and bilateral salpingo-oophorectomy. One each of the right pelvic lymph nodes and external iliac lymph nodes showed metastatic deposits. The patient was staged as FIGO stage I [AJCC TNM staging (8th Edition): pT2b pN1]. Post 6 months follow up there is no evidence of any recurrence.

## Introduction

Clear cell adenocarcinoma of cervix is a rare neoplasm, especially in younger patients. It accounts for 4-9% of all uterine cervical adenocarcinoma^1,2^.A high significant association between diethylstilbesterol (DES) in utero exposure and subsequent clear cell adenocarcinoma of cervix in young women in later life was published in 1971^3^. But cases have been reported without exposure of DES in both young and older women likewise. It is important to note that pediatric patients tend to have a higher incidence of late-stage (stage III and IV) disease compared to adult patients diagnosed with this condition^4^. Typically, patients present with symptoms such as abnormal vaginal bleeding (including premenarchal, intermenstrual, postcoital, or postmenopausal), abnormal vaginal discharge, abdominal pain, or swelling in the lower extremities^5^. We report a case of clear cell adenocarcinoma in a young woman without any history of in -utero exposure of DES with the possible morphological mimics.

## Case presentation

**Figure 1:**
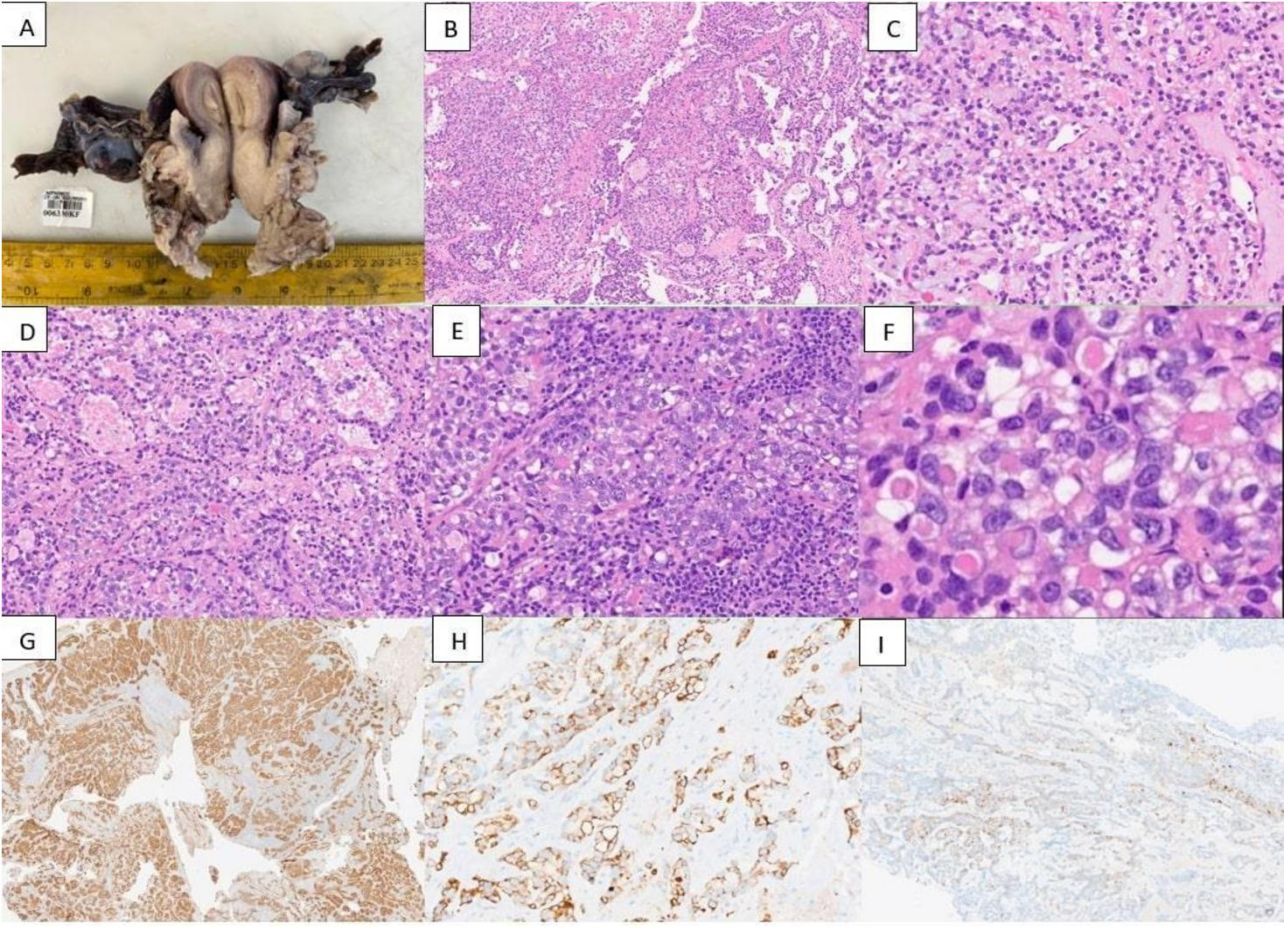
Gross image shows a grey-white solid tumor involving cervix (A). Microscopic images of clear cell carcinoma (B-F) show an infiltrating tumor disposed in tubulo-cystic pattern and papillary pattern (B, H&E, ×40), tubulocystic areas, tumor cells exhibiting clear to eosinophilic cytoplasm with nuclear hobnailing. (C,D H&E,×200), a dense lymphocytic infiltrate seen focally (E, H&E, ×200), hyaline globules (F, H&E, ×400). Microscopic images of immunohistochemical results (G-I) .Tumor cells are diffusely positive for pancytokeratin (G, immuoperoxidase,×40), CK7 (H, immuoperoxidase,x200), and Napsin A (I, immuoperoxidase,x40).

An 18-year-old girl presented with complaints of irregular on and off per vaginum (PV) bleeding and watery cervical discharge for a year. The patient had no relevant personal or family history. The patient was treated with progesterone 5 mg in a different hospital for same complaints but was not relieved of her symptoms. Pelvic MRI revealed a heterogeneously enhancing exophytic mass arising from the anterior cervical lip, measuring 4.2 x 7.3 x 4.5 cm. The uterus and ovaries were normal, and an enlarged indeterminate left external iliac node was seen measuring 2 x 1.3 cm. A cervical biopsy was performed which showed a tumor disposed of in tubule-cystic, solid nested and focal papillary pattern separated by fibrovascular septa (Figure 1A). The tumor cells show mild to moderate anisonucelosis, hyperchromatic to vesicular chromatin, occasional prominent nucleoli, and a moderate amount of eosinophilic to clear cytoplasm. The tubulo-cystic areas show nuclear hob nailing. Intervening stroma was hyalinized and showed myxoid change at places. (Figure 1B-E) Areas of necrosis were noted.

Intracytoplasmic hyaline globules were evident. (Figure 1F) Dense lymphoplasmacytic inflammatory infiltrates are seen within fibrous septa (Figure 1E) Foam cells aggregates were noted. Scattered mitoses were noted. Based on morphology a differential diagnosis of clear cell adenocarcinoma, germ cell tumor (GCT) (yolk sac tumor), and adenocarcinoma with clear cell change was considered and the tissue was subjected to immunohistochemistry (Table 1). On immuno-histochemistry, tumor cells were diffusely positive for AE1/AE3(pan-cytokeratin), CK7, and Napsin A (focal strong) while were negative for ER (ruling out endometrioid adenocarcinoma), SALL4 (ruling out GCTs), CEA, and p53 (wild type). p16 showed mosaic type of staining ruling out HPV association. Diagnosis of clear cell adenocarcinoma of the cervix (CCAC) was rendered based on histopathological and immunohistochemical findings.

Subsequently, a thoracoabdominal CT scan was performed for metastatic workup. No hepatic, pulmonary or skeletal metastasis was not found. The patient underwent total abdominal hysterectomy with bilateral salpingo-oophorectomy (TAH-BSO) and bilateral pelvic lymph node dissection. Bilateral ovaries, uterus, parametrium, and bilateral fallopian tubes were uninvolved. One each of the right pelvic lymph nodes and external iliac lymph nodes showed metastatic deposits. The patient was staged FIGO stage I [AJCC TNM staging (8th Edition): pT2b pN1].

Adjuvant radiotherapy was planned for this patient. Patient had received adjuvant chemoradiation (6cycles of Cisplatin and External beam radiotherapy 50Gy/25#) followed by vaginal cuff brachytherapy (6Gy/2#). At last follow up, after 1 year and 2 months of completing treatment patient is doing well without any complaints.

## Discussion

In-utero DES exposure was a major risk factor associated with the development of clear cell adenocarcinoma of the cervix as described by Herbst AL et al in their study in 1971.

Subsequently, after the ban of DES, Herbst reviewed 705 cases of cervical and vaginal clear cell adenocarcinoma, of which 30% cases had no exposure history of DES^6^. The pathogenesis of non-DES exposure associated with clear cell adenocarcinoma of the cervix is unclear till now and the causal factors are postulated to be similar to the conventional carcinomas.

Clear cell adenocarcinoma of cervix cases shows two peaks of incidences according to multiple studies^7^. The first peak is found between 17 to 37 years of age (mean age, 26 years), while the second peak is between 44 to 88 years of age (mean age, 71 years).

Clear cell adenocarcinoma of the cervix may present as an exophytic lesion, an endophytic lesion (with a grossly normal-appearing cervix), or a barrel-shaped cervix. During physical examination, the lesions can vary in appearance, sometimes presenting as raised nodular red lesions or small punctate ulcers. On average, the size of the lesion at diagnosis is 3.3 cm^8^.

**Table 1:** Possible differential diagnosis with immunohistochemistry and molecular alterations to differentiate the entities.

Tumor types						Immunohistochemistry						Molecular findings
	Napsi n	HNF 1β	Vimentin	ER	PR	p53	SA LL4	Glypican 3	AFP	WT 1	PAX8	
Clear cell adeno-carcinoma	+	+				Wild type, usually		_/+			+/_	PI3K, ARID1 A, MMR genes
Yolk sac tumor	+	+				Wild type, usually	+	+	+			Usually isochromosome 12p
High-grade Serous carcinoma with clear cell changes	_	_	_	+ (patchy to diffuse)	+ (patchy to diffuse)	Mutant type	_	_	_	+	+	p53, BRCA 1, BRCA 2, HRD with genomic instability
Endometrioid carcinoma with clear cell changes	_	_	+	+	+	Wild type, usually	_	_	_	_	_	PTEN, β- catenin, ARID1 A, MMR genes

The microscopic patterns of CCAC include solid, tubule-cystic and papillary patterns, papillary pattern is the least common. The tubule-cystic pattern is associated with most favourable outcome, followed by papillary and solid patterns, respectively. The patient in this study has complaint of irregular vaginal bleeding, which is the most common initial symptom. Because of this non-specific clinical finding, diagnosis is often delayed. Moreover, the age and microscopic images can be a confounding factor. The presence of cystic spaces with hyaline globules may suggest a diagnosis of yolk sac tumor in young patients. In elderly population the differential diagnosis can include serous/ endometrioid carcinoma with clear cell change or even a squamous cell/ adenocarcinoma of cervix with clear cell change. Table 1 shows the possible differential diagnosis with immunohistochemical markers to differentiate the entities.

Treatment in young patient with this condition usually includes hysterectomy with fertility preservation. However, this case had already presented with lymph node metastasis which was confirmed on frozen and hence was subjected to TAH-BSO with lymph node dissection. Treatment strategy is not well described in CCAC because of relative paucity of literature and rarity of cases. For early-stage disease, surgery is the definitive treatment^9^. Most studies agree that clear cell carcinoma is a tumor linked to lymphovascular space invasion and lymph node metastases, with pelvic lymph node involvement observed in up to 25% of cases. Similarly, Stolnicu et al. reported lymphovascular invasion in 31% of cases and lymph node metastases in 24.1%. Additionally, 10.3% of cases were associated with abdomino-pelvic metastases, 32.8% experienced recurrences, and 19% succumbed to the disease^10^. Important negative prognostic factors are; a tumor more than 4 cm in size, positive lymph node metastasis, and advanced stages along with few histopathological findings like nuclear atypia, high mitotic count and solid growth pattern^1,9^. The FIGO stage and pelvic node status are the most important prognostic factors for progression-free survival and overall survival in these patients^11^. Key risk factors for recurrence include positive parametrial extension, positive pelvic lymph nodes, and positive vaginal margins. For patients with these high-risk factors, chemoradiation is the preferred postoperative adjuvant treatment^11^.

## Conclusion

Etiopathogenesis of non-HPV-related cervical cancer is still unknown and it has mainly nonspecific clinical features that cause misdiagnosis of this entity in many times. As it can affect the pediatric population and adolescents, fertilizing preservation becomes an important consideration in the management of these patients, So, early diagnosis by clinicopathological correlation is needed for better management and disease-free survival for the patients with CCAC.
